# Misdiagnosis of non-tuberculous mycobacteria as tuberculous by the GeneXpert MTB/RIF Ultra: Fact or fiction?

**DOI:** 10.4102/sajid.v39i1.576

**Published:** 2024-03-29

**Authors:** Christoffel J. Opperman, Zethembe Ntshangase, Minkie Gumede, Sarishna Singh, Yonas Ghebrekristos, Rob Warren, Wynand Goosen

**Affiliations:** 1 National Health Laboratory Service, Green Point TB-Laboratory, Cape Town, South Africa; 2 SAMRC Centre for Tuberculosis Research, Division of Molecular Biology and Human Genetics, Faculty of Health Science, Stellenbosch University, Cape Town, South Africa; 3 Division of Medical Microbiology, Department of Pathology, Faculty of Health Science, University of Cape Town, Cape Town, South Africa; 4 Faculty of Health Science, University of Cape Town, Cape Town, South Africa

## Introduction

GeneXpert detects *Mycobacterium tuberculosis* complex (MTBC) at low bacterial loads, exhibiting high sensitivity and specificity for smear-negative samples.^1^ It was reported that GeneXpert^®^ MTB/RIF (Sunnyvale, CA, United States [US]) incorrectly identified several American Type Culture Collection group (ATCC) non-tuberculous mycobacteria (NTM) (*Mycobacterium abscessus, Mycobacterium marinum, Mycobacterium smegmatis, Mycobacterium phlei* and *Mycobacterium aurum*) as MTBC when high bacterial loads [10^6^ colony forming units/millilitre (CFU/mL)] were present.^2^ In these studies, high (> 30) cycle threshold values for probe E resulted in *M. abscessus* and *M. smegmatis* flagging as rifampicin resistant.^2^ Similar findings were noted between GeneXpert^®^ MTB/RIF probe A and *M. intracellulare* that produced positive signals, most likely because of a mismatch between the probe and the deoxyribonucleic acid (DNA) target.^3^ The outcomes and prognosis for a patient receiving anti-tuberculosis (TB) regimens when harbouring a clinically significant NTM is potentially poor, with a high risk of treatment failure.^2^ Hence, this study aimed to investigate whether the GeneXpert^®^MTB/RIF Ultra (Ultra, Sunnyvale, CA, USA) assay could yield true-negative results on high bacterial load dilutions of NTM commonly occurring in clinical samples. In addition, a secondary objective assessed the presence of the four probes covering the 81-base pair core region within the ribonucleic acid (RNA) polymerase β-subunit gene (*rpoB*), as identified by the Xpert Ultra assay, among NTM and among NTM and a selection of other bacteria.

## Methods

A total of 12 NTM species obtained from clinical samples, and 11 other bacteria from an ATCC group were included in the study (Table 1). Pure NTM organisms were cultured on Löwenstein–Jensen media (Thermo Scientific™) slants at 35 °C ± 2 °C between 1 week and 4 weeks, dependent on the different NTM growth rates. A Ziehl-Neelsen stain was done on all NTM cultures to check for acid-fast bacilli and rule out contamination. Identification of NTM species was confirmed with Genotype^®^ Mycobacterium CM and AS (Bruker, Billerica, MA, US) line probe assays (LPA). Bacteria, distinct from NTM, encompassed in the investigation were cultured on 2% blood agar and boiled blood agar, with an incubation period of 24 h - 48 h (Table 1). The NTM and other bacteria were collected from the solid media and homogenised in sterile water to reach the desired 0.33 McFarland, representing 1 × 10^8^ CFU/mL. Xpert Ultra testing was performed in duplicate according to the manufacturer’s instructions.^4^

**TABLE 1:** GeneXpert MTB/RIF Ultra (cycle thresholds) performend on non-tuberculous mycobacteria and a selection of other bacteria.

Organism	Sample processing control††	Insertion sequences IS*1081*/IS*6110*†	Probe†				Probe check control††	Test result
			rpoB1	rpoB2	rpoB3	rpoB4		
*Mycobacterium lentiflavum* ‡	26.1	0	0	34.0	0	0	Pass	MTBC not detected
*Mycobacterium chelonae*	25.5	0	0	0	0	0	Pass	MTBC not detected
*Mycobacterium scrofulaceum*	24.4	0	0	26.0	0	0	Pass	MTBC not detected
*Mycobacterium avium*	24.4	0	0	32.0	0	0	Pass	MTBC not detected
*Mycobacterium gordonae*	25.8	0	0	23.7	0	33.6	Pass	MTBC not detected
*Mycobacterium palustre* §	22.3	0	0	18.8	34.1	0	Pass	MTBC not detected
*Mycobacterium kansasii*	22.0	0	38.5	18.6	0	37.8	Pass	MTBC not detected
*Mycobacterium szulgai*	24.9	0	37.8	20.1	0	0	Pass	MTBC not detected
*Mycobacterium mucogenicum* ‡	23.8	0	0	24.6	0	0	Pass	MTBC not detected
*Mycobacterium septicum* §	23.8	0	0	29.6	0	0	Pass	MTBC not detected
*Mycobacterium fortuitum group*	24.1	0	0	0	0	0	Pass	MTBC not detected
*Mycobacterium abscessus* subsp. *bolletii*§	23.4	0	0	36.1	0	0	Pass	MTBC not detected
*Klebsiella pneumonia* ATCC 700603	25.3	0	0	0	0	0	Pass	MTBC not detected
*Pseudomonas aeruginosa* ATCC 27853	25.9	0	0	0	0	0	Pass	MTBC not detected
*Staphylococcus aureus* ATCC 25923	26.7	0	0	0	0	0	Pass	MTBC not detected
*Escherichia coli* ATCC 25933	26.1	0	0	0	0	0	Pass	MTBC not detected
*Salmonella typhimurium* ATCC 8382	26.3	0	0	0	0	0	Pass	MTBC not detected
*Listeria monocytogenes* ATCC 11916	26.1	0	0	0	0	0	Pass	MTBC not detected
*Enterococcus faecalis* ATCC 51299	25.3	0	0	0	0	0	Pass	MTBC not detected
*Haemophilus influenzae* ATCC 49247	26.3	0	0	0	0	0	Pass	MTBC not detected
*Streptococcus pyogenes* ATCC 19615	27.1	0	0	0	0	0	Pass	MTBC not detected
*Streptococcus agalactiae* ATCC 55617	27.0	0	0	0	0	0	Pass	MTBC not detected
*Streptococcus pneumonia* ATCC 49619	23.2	0	0	0	0	0	Pass	MTBC not detected
*Mycobacterium tuberculosis* complex (H37Rv)¶	29.5	16.2	17.3	17.4	18.0	20.3	Pass	MTBC detected

ATCC, American Type Culture Collection; IS, Insertion sequence; MBTC, *Mycobacterium tuberculosis* complex; rpoB, RNA polymerase (β subunit).

† , GeneXpert®MTB/RIF Ultra is a real-time, semi-quantitative, nested PCR platform (40 cycles) with four probes (rpoB1–4) that covers the 81 base pair core region of the RNA polymerase β-subunit gene (*rpoB*) to detect rifampicin susceptibility. In addition, the current version has two insertion sequences (IS*1081* and IS*6110*) that detect *the Mycobacterium tuberculosis* complex. The sloppy molecular beacons (rpoB1–4) hybridise with the *rpoB* gene before they are melted off the amplicon targets. Melting temperature analysis allows for the detection of rifampicin susceptibility, including heteroresistance. In contrast, the previous version (GeneXpert^®^MTB/RIF assay) identified *Mycobacterium tuberculosis* complex when at least two of the five molecular beacons (A–E) gave a positive signal with a cycle threshold (Ct) value of ≤38 cycles. Rifampicin resistance was detected when two probes had a Ct delta of ≥ 4.1 (delayed probe binding) or when at least one probe showed no signal (probe dropout).^8^

‡ , Genotype®Mycobacterium AS line probe assays were implemented to identify the *Mycobacterium* species.

§ , Selected mycobacteria were confirmed with Sanger sequencing of the heat shock gene (*hsp65*), submitted to the Central Analytical Facility, Stellenbosch University.

¶ , The American Type Culture Collection strain (H37Rv) *Mycobacterium tuberculosis* was used as the positive control, and sterile water was a DNA-absent control.

†† , Sample processing and probe check controls confirm a valid test.

## Results

*Mycobacterium tuberculosis* complex was not detected in any NTM or other bacterial samples evaluated in the cohort selected (Table 1). Furthermore, the rpoB2 probe signal was noticed in 10/12 NTM samples and 0/11 from other bacteria. From the 10 NTM with positive rpoB probe signals, 6 showed an isolated rpoB2 positive probe, while combinations ([rpoB1 + rpoB2]; [rpoB2 + rpoB3]; [rpoB2 + rpoB4]; [rpoB1 + rpoB2 + rpoB4]) together with other rpoB probes were positive in 4 isolates (Table 1).

## Discussion

Non-tuberculous mycobacteria infection is an emerging disease with complex and extended antibiotic treatment regimens.^5^ A 5-year all-cause mortality study of > 9000 patients with NTM pulmonary disease estimated a case fatality rate of 27% (95% confidence interval: 21.3–37.8).^6^ Clinical and radiological features of pulmonary NTM disease can appear similar to MTBC disease.^7^ Therefore, accurate diagnosis of NTM disease remains essential. Xpert Ultra is a front-line diagnostic test for identifying TB in many countries, such as South Africa. The current study confirms the specificity of Xpert Ultra to accurately distinguish between NTM and MTBC, providing confidence in diagnosing TB and starting treatment if clinically indicated.

The current South African TB diagnostic algorithm places its primary emphasis on detecting MTBC at the time of a patient’s initial presentation. Consequently, it may overlook the presence of NTM unless cultures are specifically conducted for further LPA analysis. An Xpert Ultra MTBC-negative sample with a positive *rpoB* gene signal could suggest an NTM (Table 1). However, the absence of positive rpoB probes does not preclude the presence of NTM. In circumstances where MTBC is not present, the utilisation and interpretation of rpoB signals on Xpert Ultra remain unclear. This could be relevant on a case-by-case basis in recurrent Xpert Ultra-negative patients with constitutional symptoms and radiological chest X-ray changes, such as cavities, a scenario sometimes encountered in the clinical setting. Additional investigation is imperative with a more comprehensive exploration of these intriguing findings within the realm of NTM/MTBC diagnostics. It remains uncertain whether larger NTM cohorts will demonstrate similar results or if these findings hold any relevance within existing diagnostic algorithms. Further research is essential to shed more light on this matter. In addition, we acknowledge that future investigations should incorporate sputum samples to better reflect the *in vivo* environment. Lastly, the question of ‘… are we doing everything with the data we have …’ was asked at the PathRed 2023 conference in Johannesburg, South Africa.^9^ The current approach suggested in this study allows us to do more with the data we already have, providing the opportunity for deeper data mining of our TB diagnostic results.

## References

[1] Dorman SE, Schumacher SG, Alland D, et al. Xpert MTB/RIF Ultra for detection of *Mycobacterium tuberculosis* and rifampicin resistance: A prospective multicentre diagnostic accuracy study. Lancet Infect Dis. 2018;18(1):76–84. 10.1016/S1473-3099(17)30691-6 29198911 PMC6168783

[2] Pang Y, Lu J, Su B, Zheng H, Zhao Y. Misdiagnosis of tuberculosis associated with some species of non-tuberculous mycobacteria by GeneXpert MTB/RIF assay. Infection. 2017;45(5):677–681. 10.1007/s15010-017-1044-x 28707184

[3] Tang Y, Yu J, Yang G, et al. Probe A shown in the GeneXpert MTB/RIF assay during the detection of *Mycobacterium intracellular* infections. Diagn Microbiol Infect Dis. 2021;99(2):115243. 10.1016/j.diagmicrobio.2020.115243 33130506

[4] Xpert® MTB/RIF Ultra [homepage on the Internet]. [cited 2023 Sept 18]. Available from: https://www.cepheid.com/en-GB/tests/tb-emerging-infectious-diseases/xpert-mtb-RIF-ultra.html

[5] Daley CL, Iaccarino JM, Lange C, et al. Treatment of non-tuberculous mycobacterial pulmonary disease: An official ATS/ERS/ESCMID/IDSA clinical practice guideline. Eur Respir J. 2020;56(1):2000535. 10.1183/13993003.00535-2020 32636299 PMC8375621

[6] Diel R, Lipman M, Hoefsloot W. High mortality in patients with *Mycobacterium avium* complex lung disease: A systematic review. BMC Infect Dis. 2018;18(1):206. 10.1186/s12879-018-3113-x 29724184 PMC5934808

[7] Liu Q, Du J, An H, et al. Clinical characteristics of patients with non-tuberculous mycobacterial pulmonary disease: A seven-year follow-up study conducted in a certain tertiary hospital in Beijing. Front Cell Infect Microbiol. 2023;22(13):1205225. 10.3389/fcimb.2023.1205225 PMC10325861 37424783

[8] Lawn SD, Nicol MP. Xpert®MTB/RIF assay: Development, evaluation and implementation of a new rapid molecular diagnostic for tuberculosis and rifampicin resistance. Future Microbiol. 2011;6(9):1067–1082. 10.2217/fmb.11.84 21958145 PMC3252681

[9] Grant Theron. Doing better with what we already have: Improving tuberculosis confirmatory and drug susceptibility testing (a laboratory perspective). PathRed Conference; 2023 Sept 01; Johannesburg.

